# Advancing hepatology through innovation and integration: *iLIVER*'s vision for 2026

**DOI:** 10.1016/j.iliver.2026.100224

**Published:** 2026-02-06

**Authors:** Zu-Chao Du, Ke-Chun Wang, Ming-Da Wang, Lei Cai, Jian-Hong Zhong, Tian Yang

**Affiliations:** a Department of Hepatobiliary Surgery, The First Affiliated Hospital, Henan University, Kaifeng 475000, Henan, China; b Department of Hepatobiliary Surgery, Eastern Hepatobiliary Surgery Hospital, Naval Medical University, Shanghai 200438, China; c Institute of Hepatopancreatobiliary Surgery, Chongqing General Hospital, Chongqing University, Chongqing 401147, China; d Department of Hepatobiliary Surgery, Affiliated Tumor Hospital of Guangxi Medical University, Nanning 530021, Jiangsu, China

**Keywords:** Hepatocellular carcinoma, Artificial intelligence, Liquid biopsy, Conversion therapy, Metabolic dysfunction-associated steatotic liver disease, Precision medicine

Liver diseases continue to impose a substantial and evolving burden on global health up to 2026, characterised by a wide clinical spectrum and increasing therapeutic complexity. Liver cancer remains the sixth most common malignancy and the third leading cause of cancer-related mortality worldwide, with hepatocellular carcinoma (HCC) accounting for nearly 75% of all primary liver cancers.^1^ Although sustained viral hepatitis control programs have stabilized or reduced virus-related HCC incidence in many regions, its epidemiological profile is undergoing a marked transition, with metabolic dysfunction-associated steatotic liver disease (MASLD) increasingly contributing to HCC cases, driven by global obesity and metabolic syndrome epidemics.^2^ In parallel, advances in molecular science, artificial intelligence (AI)-driven diagnosis and precision liver surgery, and systemic immunotherapy are reshaping liver disease management.^3^ These developments highlight the need to refine strategies for conversion therapy, adjuvant treatment, and recurrence prevention, and underscore the urgency of coordinated research efforts that translate biological and technological innovation and empirical management into sustained improvements in patient outcomes.

The year 2025 marked remarkable milestones for *iLIVER*. The journal received 138 submissions, a 58.6% increase compared with the previous year, and published 40 high-quality papers with an average review turnaround of 33 days. Since 2022, *iLIVER* has accumulated 350 citations, with 201 recorded in 2025, while annual article usage reached 10,1515, reflecting 53% year-on-year growth. The journal was indexed in PMC, Scopus, DOAJ, OARL, and the Chinese Science and Technology Core Journal database, and made a notable appearance at the 34th Asian Pacific Association for the Study of the Liver Annual Meeting (APASL 2025). Notably, 31 members of the editorial boards were recognized among the top 2% of scientists worldwide in 2025.

Standing at the threshold of 2026, hepatology is undergoing a profound transformation from traditional empirical medicine toward biology-driven, multimodal-integrated precision care. Over the past year, under the leadership of Academician Jia-Hong Dong, *iLIVER* has published a series of practice-changing contributions with the potential to transform clinical practice, spanning artificial intelligence, liquid biopsy, and conversion therapy. In the domain of artificial intelligence and radiomics, Zhang and colleagues pioneered a multimodal framework integrating traditional Chinese medicine tongue-based assessment with clinical biomarkers, enabling cardiovascular risk stratification in MASLD patients through deep learning algorithms.^4^ Complementing these AI advances, Peng and colleagues constructed a multi-phase magnetic resonance imaging (MRI)-based radiomics model that significantly improved the preoperative prediction of microvascular invasion in HCC, a critical determinant of surgical planning and oncological outcomes.^5^ In the field of liquid biopsy and molecular diagnostics, Liu and colleagues established bile cell-free DNA (cfDNA) as a superior substrate compared with plasma for somatic mutation detection in cholangiocarcinoma, thereby expanding the diagnostic armamentarium for early detection and molecular characterization of this aggressive malignancy.^6^ Regarding clinical surgery and therapeutic innovation, Wang and colleagues systematically delineated the philosophical and technical underpinnings of precision liver surgery, advocating for visualizability, quantifiability, and controllability as the tripartite foundation guiding contemporary surgical practice.^7^ Building on these surgical principles, Kawaguchi emphasized the clinical value of optimizing minimally invasive anatomical hepatectomy through the Laennec capsule approach, providing practical guidance for precise parenchymal transection.^8^ In the therapeutic domain, Chen and colleagues reported encouraging outcomes demonstrating that hepatic arterial infusion chemotherapy combined with immune checkpoint inhibitors represents a safe and effective strategy for converting initially unresectable HCC to surgical candidacy.^9^ Additionally, Sun and colleagues proposed a conceptual framework for individualized MASLD management that acknowledges disease heterogeneity by distinguishing liver-dominant phenotypes from systemic cardiometabolic variants, offering guidance for precision therapeutic approaches.^10^ Collectively, these publications reflect *iLIVER*'s commitment to advancing hepatology research from fundamental science to clinical translation, driving progress in hepatological practice and therapeutic innovation.

In the upcoming phase, *iLIVER* will continue its journey to explore emerging frontiers in hepato-pancreato-biliary diseases, reshaping the landscape of precision healthcare through the integration of surgical innovation and multimodal therapeutic paradigms (Fig. 1). In surgical oncology, the definition of resectability is evolving from a purely anatomical assessment toward a deep collaborative framework governed by the multidisciplinary team. To minimize futile treatment, AI-assisted surgical decision-making will be anchored in the three essential elements of precision liver surgery, namely leision clearance, liver preservation, and damage control. Addressing the persistent challenge of postoperative recurrence, liquid biopsy-based cfDNA mutation and methylation profiling enables high-sensitivity detection of minimal residual disease, offering transformative potential for monitoring the evolutionary trajectory from chronic liver disease to hepatocellular carcinoma. In basic and translational research, we seek to decipher the immune microenvironment remodeling mechanisms governing the transition from MASLD to HCC, focusing on exosome-mediated signaling within fibrotic niches and the orchestration of immunosuppressive states.

**Fig. 1:**
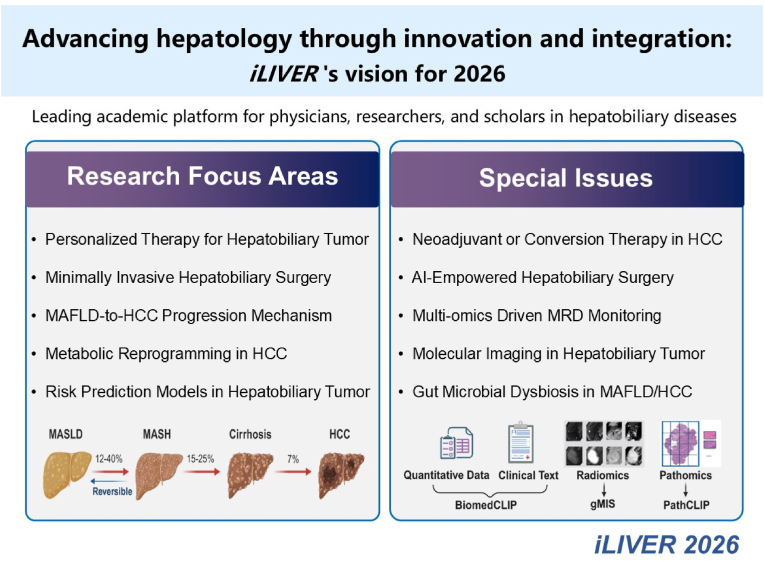
*iLIVER* 2026: Research Focus Areas & Special Issues. Created in BioRender. Du, Z. (2026) https://BioRender.com/ipmdngp. Abbreviation: HCC, Hepatocellular carcinoma; MAFLD, metabolic dysfunction-associated fatty liver disease; MRD, minimal residual disease; AI, artificial intelligence.

In response to these emerging themes, *iLIVER* has launched four focused special issues addressing critical frontiers in hepatobiliary medicine. Newly introduced collections examine neoadjuvant or conversion therapy in hepatobiliary cancer, guest edited by Prof. Jian-Hong Zhong and Prof. Tian Yang (https://www.sciencedirect.com/special-issue/329035/neoadjuvant-or-conversion-therapy-in-hepatobiliary-cancer), and metastatic liver cancer: global innovations in multidisciplinary diagnosis and treatment, guest edited by Prof. Tao Peng (https://www.sciencedirect.com/special-issue/329271/metastatic-liver-cancer-global-innovations-in-multidisciplinary-diagnosis-and-treatment). Continuing from 2024, collections on artificial intelligence in liver diseases (https://www.sciencedirect.com/special-issue/317553/artificial-intelligence-in-liver-diseases) and Laennec capsule-based anatomical procedures in hepatobiliary surgery (https://www.sciencedirect.com/special-issue/317074/laennec-capsule-based-on-anatomical-procedures-in-hepatobiliary-surgery) remain open for submissions. Collectively, these collections reflect *iLIVER's* commitment to integrating technological innovation with precision oncology and surgical advancement, fostering international collaboration in hepatobiliary science.

With this foundation of achievement, *iLIVER* will continue to build upon the academic excellence of global hepatology and China's research strengths in the field. The journal maintains its dedication to upholding rigorous peer review standards and optimizing publication processes. Through these efforts, *iLIVER* strives to further develop as an outstanding academic journal and distinguished platform for international scholarly exchange, advancing the frontiers of hepatobiliary science and clinical practice worldwide.

## CRediT authorship contribution statement

**Zu-Chao Du:** Conceptualization, Data curation, Writing – original draft. **Ke-Chun Wang:** Conceptualization, Writing – original draft. **Ming-Da Wang:** Conceptualization, Data curation, Writing – review & editing. **Lei Cai:** Conceptualization, Formal analysis, Writing – review & editing. **Jian-Hong Zhong:** Conceptualization, Writing – review & editing. **Tian Yang:** Conceptualization, Data curation, Writing – review & editing.

## Informed consent

Not applicable.

## Ethics statement

Not applicable.

## Data availability statement

Not applicable as no data sets were generated or analyzed.

## Declaration of Generative AI and AI-assisted technologies in the writing process

Not applicable.

## Funding

None.

## Declaration of competing interest

The author Tian Yang is Executive Editor-in-Chief and Lei Cai is an Executive Assistant Editor for this journal and they were not involved in the editorial review or the decision to publish this article.
